# Implant Therapy and Frailty: Outcomes, Risk Stratification, and Decision Algorithms

**DOI:** 10.7759/cureus.97105

**Published:** 2025-11-17

**Authors:** Chinmoy Sikdar, Shubham K Srivastava, Akshim Rana, Shitij Srivastava, Pankaj Dewanjee

**Affiliations:** 1 Maxillofacial Prosthodontics and Implantology, Sardar Patel Post Graduate Institute of Dental and Medical Sciences, Lucknow, IND; 2 Periodontology and Implantology, Regional Dental College, Guwahati, IND

**Keywords:** aging population, dental implants, frailty, implant therapy, prosthodontics

## Abstract

As global populations transition into an era of super-aging, implant therapy increasingly extends to older adults with multiple comorbidities and functional decline. Traditional reliance on chronological age as a determinant of treatment eligibility is gradually being replaced by a more holistic understanding of frailty, a multidimensional measure of biological resilience and vulnerability. Recognizing frailty as a dynamic continuum rather than a fixed state can reshape decision-making in implant dentistry, emphasizing patient-centered care over procedural ambition. Contemporary evidence suggests that while implant survival in older adults remains favorable, outcomes are significantly influenced by frailty status, systemic health, and the capacity for long-term maintenance. Incorporating brief frailty assessment tools such as the Clinical Frailty Scale (CFS) or Edmonton Frail Scale (EFS) into preoperative planning enables risk stratification, better communication with caregivers and physicians, and alignment of treatment goals with patient quality of life. This editorial advocates for a paradigm shift from age-based to frailty-based decision algorithms, promoting minimally invasive protocols, simplified prosthetic designs, and proactive maintenance strategies. Integrating geriatric principles into implant therapy not only enhances clinical predictability but also reinforces ethical stewardship in delivering personalized, sustainable care for the aging population.

## Editorial

The field of dental implantology has evolved profoundly over the past three decades, transforming from a selective surgical procedure to a widely adopted restorative modality. As life expectancy continues to increase globally, the dental community now serves an expanding demographic, the super-aging population defined by individuals aged 80 years and older seeking implant-supported prosthetic rehabilitation to restore mastication, phonetics, and esthetics. However, this clinical opportunity is accompanied by a growing challenge: reconciling restorative ambition with biological vulnerability. In this context, frailty, not chronological age, has emerged as the critical determinant of treatment outcomes, encompassing the physiological capacity to withstand surgical stress, recover effectively, and maintain long-term oral function [1].

From chronological age to frailty: redefining risk in implant dentistry

Historically, advanced age was considered a limiting factor for implant therapy due to presumed compromises in bone quality, healing potential, and systemic resilience. However, recent systematic reviews have shown that age per se does not significantly affect early implant losses or overall survival rates when appropriate clinical protocols are applied [1]. Implant outcomes in elderly patients are largely comparable to those in younger cohorts, provided that treatment planning and maintenance are individualized. The decisive factor is frailty, a multidimensional construct reflecting cumulative physiological decline that reduces the body’s ability to adapt to stressors such as surgery, infection, or prosthetic loading [2].

Frailty differs from chronological aging in that it is dynamic and modifiable. A patient may appear medically stable under baseline conditions but experience disproportionate decline after even minor surgical trauma. Thus, implant clinicians must move beyond age-based exclusion criteria toward functional and resilience-based evaluation [3]. Recognizing and quantifying frailty enables clinicians to stratify surgical risk, optimize preoperative preparation, and design treatment plans that match the individual’s physiological reserve rather than their age.

Clinical understanding and assessment of frailty

Frailty is clinically assessed using validated tools that capture physical, cognitive, and psychosocial parameters. The Clinical Frailty Scale (CFS), developed from the Cumulative Deficit Model, categorizes patients from 1 (very fit) to 9 (terminally ill) based on overall health status, comorbidities, and functional independence [3]. This scale has proven highly practical in medical and dental settings, allowing quick chairside screening without sophisticated equipment.

Other models, such as the Fried Phenotype, define frailty through five criteria, namely, weight loss, exhaustion, weakness, slowness, and low activity, while the Edmonton Frail Scale (EFS) incorporates cognition and social support [4]. Importantly, frailty is not an irreversible condition. Interventions such as exercise therapy, nutritional supplementation, and medical optimization can partially reverse frailty and improve surgical outcomes [2]. Hence, identifying frailty preoperatively should not automatically preclude implant therapy; rather, it allows for targeted “prehabilitation” and interdisciplinary management.

Implant survival and frailty-linked variability

Long-term data reveal that dental implants in older adults demonstrate high survival rates, frequently exceeding 90% over 10 years [1,4]. Yet when results are stratified by frailty rather than age, outcome variability becomes evident. Frail individuals often exhibit higher rates of early failure, peri-implantitis, and prosthetic complications due to compromised vascularity, immune dysfunction, sarcopenia, and impaired manual dexterity [4]. Additionally, cognitive decline and dependency may reduce adherence to oral hygiene and maintenance regimens, leading to biofilm accumulation and biological failure.

Importantly, successful osseointegration in frail patients does not guarantee functional longevity. The sustainability of prosthetic comfort and hygiene relies heavily on caregiver involvement and recall support. Thus, frailty affects not only the surgical outcome but also the long-term usability and maintenance of implant restorations. Understanding these limitations helps clinicians align treatment goals with realistic expectations of function and comfort [5].

Frailty-based risk stratification for treatment planning

Integrating frailty assessment into implant planning transforms it from a theoretical concept into a clinical decision tool. The CFS provides a practical stratification framework.

CFS 1-3 (Fit-Managing Mild Stressors)

Conventional implant protocols are appropriate, with standard healing and loading times.

CFS 4-5 (Vulnerable-Mildly Frail)

This involves the use of minimally invasive techniques such as flapless guided surgery, short implants, or delayed loading to reduce surgical trauma.

CFS 6-7 (Moderately Frail)

The intervention must be limited to simple, functionally essential restorations, such as two-implant overdentures or mini-implants, to balance benefit and risk.

CFS ≥ 8 (Severely Frail/Terminally Ill)

Surgical implant therapy must be avoided; instead, emphasis should be placed on removable, tissue-supported prostheses that prioritize comfort and hygiene [3].

This stratified approach allows clinicians to transform frailty from an exclusion criterion into an inclusion framework, enabling proportionate, patient-centered decision-making that prioritizes safety, function, and dignity.

Minimally invasive and digital innovations for the frail elderly

Technological advances have revolutionized implant surgery for older adults. Cone-beam computed tomography (CBCT), digital intraoral scanning, and computer-guided implant placement allow for precision, predictability, and reduced operative stress. Minimally invasive protocols, such as flapless or guided placement, shorten surgical duration, minimize tissue trauma, and improve postoperative comfort [1,5].

Moreover, the availability of short and ultrashort implants reduces the need for bone augmentation, which is particularly advantageous in patients with limited healing capacity. Prosthetic designs emphasizing simplicity, such as screw-retained single crowns or locator-retained overdentures, facilitate hygiene and retrievability, especially when caregiver assistance is necessary [4]. These innovations enable clinicians to achieve functional rehabilitation while respecting the biological constraints of frailty.

Ethical dimensions: balancing rehabilitation with life expectancy

Ethical considerations form the cornerstone of implant therapy in the frail elderly. A complex, multistage treatment may offer limited benefit if the patient’s functional lifespan is short or if maintenance demands exceed their capability. The principle of proportionate rehabilitation advocates aligning treatment complexity with the expected duration of benefit and the patient’s remaining adaptive capacity [2].

Shared decision-making ensures that patients and caregivers understand procedural risks, maintenance responsibilities, and potential fallback options. For those with cognitive decline, legal guardianship and transparent documentation of consent are crucial. Ethical implantology, therefore, must prioritize safety, comfort, and dignity over technical perfection [5].

Developing a frailty-based clinical decision algorithm

A structured algorithm that integrates frailty assessment into implant care enhances both predictability and safety. The process begins with an initial screening phase that involves evaluation of systemic conditions, medication profiles, and functional dependence. Frailty assessment follows, wherein the Clinical Frailty Scale (CFS) or Edmonton Frail Scale (EFS) is applied to classify the patient’s level of physiological resilience [3,4].

The subsequent phase focuses on medical optimization, during which collaboration with physicians ensures stabilization of systemic parameters before surgical intervention. Treatment planning is guided by the patient’s frailty grade, emphasizing the selection of minimally invasive surgical and prosthetic options. During the execution and support phase, short-duration, guided, and flapless protocols with meticulous aseptic measures are typically preferred. Maintenance and reassessment are characterized by regular recall visits at three- to six-month intervals, caregiver involvement, and periodic reevaluation of frailty status [3].

This structured model transforms frailty from a descriptive label into an actionable determinant that influences every phase of implant therapy.

Integrating geriatric principles into dental education and policy

Despite abundant evidence supporting frailty-based care, its application in dentistry remains inconsistent. Most prosthodontic and implant curricula still emphasize technical competence over geriatric understanding. Incorporating frailty screening, geriatric pharmacology, and interprofessional collaboration into dental education would better prepare clinicians for the realities of aging societies [2,3].

At the policy level, incorporating frailty metrics into professional implant guidelines would help standardize risk evaluation and improve outcomes. This mirrors the medical paradigm, where frailty assessment is now a prerequisite for surgical clearance in many specialties. Developing a recognized subspecialty in geriatric implantology could foster targeted research and education, bridging the gap between implant science and geriatric medicine.

Future perspectives and research directions

The intersection between frailty science and implant dentistry offers rich potential for innovation. Future clinical trials should stratify participants by frailty indices rather than chronological age to derive more meaningful evidence on implant survival, peri-implant tissue stability, and patient satisfaction [1,4]. Research into biomarkers of frailty, such as inflammatory cytokines, sarcopenic indices, or oxidative stress markers, could provide objective predictors of healing potential.

Moreover, artificial intelligence (AI) and machine learning may soon aid clinicians in predicting surgical risk based on health data, imaging, and procedural variables. These predictive models could guide treatment planning, balancing clinical benefit with physiological reserve. The integration of digital, biological, and behavioral data promises to make frailty-informed implantology more precise and personalized.

Conclusion

Frailty signifies a transformative shift in the philosophy of implant dentistry. Transitioning from an age-based exclusion to a frailty-informed inclusion model reflects both scientific advancement and ethical maturity. By adopting frailty assessment tools, minimally invasive strategies, and patient-specific maintenance protocols, clinicians can achieve safe, sustainable, and compassionate implant therapy for the super-aging population. The ultimate measure of success is not only implant survival but also the preservation of comfort, function, and dignity in later life. Embracing frailty-based algorithms ensures that every treatment reflects the patient’s life context, harmonizing clinical innovation with humane care [1-5].
